# Adsorption of insulin onto neonatal infusion sets: should intravenous administration of insulin to treat hyperglycemia in preterm babies on the NICU be proceeded by priming of the intravenous system, adding of albumin, or non-priming to get to a stable insulin dose?

**DOI:** 10.1186/s40348-022-00154-y

**Published:** 2022-12-21

**Authors:** Paola Mian, Mathieu S. Bolhuis, J. Marina Maurer, Margriet van Stuijvenberg

**Affiliations:** 1 Department of Clinical Pharmacy and Pharmacology, University Medical Center Groningen, Groningen, The Netherlands; 2 Division of Neonatology, Department of Pediatrics, Beatrix Children’s Hospital, University of Groningen, University Medical Center Groningen, Groningen, The Netherlands

## Abstract

Insulin is used to treat neonatal hyperglycaemia when blood glucose concentrations are consistently high, and to treat neonatal diabetes. Within this brief report, a review of the existing literature is conducted to determine if intravenous administration of insulin should be proceeded by priming of the intravenous system, adding of albumin, or non-priming to get a stable insulin dose. Within this literature search, we focused on experimental insulin adsorption data (in vitro studies).

## Scenario

A twin infant was born at 24 + 3 weeks gestation by vaginal delivery after spontaneous onset of labour. He was admitted to neonatal intensive care unit and received artificial ventilation. Birth weight was 0.580 kg. The infant had hyperglycemic events during an episode of sepsis. On day 35, insulin treatment was started because of persisting hyperglycemia. Insulin infusion using a solution of 0.1 EH insulin/ml in NaCl0.9%, with an initial flow of 0.21 ml/h, was started at 0.025 IU/kg/h, which was the lowest dose possible (actual body weight 0.825 kg). To ensure intravenous patency, glucose 5% at 1.0 ml/h was administered alongside. Carbohydrate intake was 8.7 mg/kg/min. For strict glucose monitoring an arterial line was given. The first 5 h glucose levels remained between 13.0 and 15.7 mmol/L (at initiation treatment was targeted to reduce the glucose level slowly but keep it above 8 mmol/L, in order to prevent hypoglycemia). Insulin treatment was stepwise adjusted to a maximum of 0.045 IU/kg/h at 48 h and stopped 7 h later after a glucose level of 1.9 mmol/L. In addition a glucose bolus was given. Two episodes of hyperglycemia occurred thereafter for which insulin treatment was given. The total duration of insulin therapy was 18 days. The diagnosis of transient neonatal diabetes could not be genetically confirmed. Follow-up at 12 months corrected age showed normal neurological development and behavior.

## Structured clinical question

In preterm babies with hyperglycemia on the NICU, should intravenous administration of insulin be proceeded by priming of the intravenous system, adding of albumin, or non-priming to get a stable insulin dose?

## Search

### Primary sources

MEDLINE, PubMed, and Embase were searched using the following search terms on 24th November 2021: Insulin AND flushing OR priming OR adsorption AND neonate OR newborn.

### Secondary sources

References of included studies were checked for relevant studies to be potentially included.

## Inclusion and exclusion criteria

After an elaborate search, no in vivo studies in preterm or term neonates with hyperglycemia were found. We therefore extended our search to all in vitro studies on recovery of insulin over time after administration over the neonatal line. We included studies that compared non–priming of the line versus priming of the line with insulin, or compared to dissolving insulin in albumin before administration over the line. No publication date restrictions were applied. The search was restricted to English studies only.

## Output

Above-mentioned search strategy resulted in, based on title and abstract, ten potentially relevant papers. After reading the full texts, eight studies [1–8] were identified as relevant to answer the clinical question. Reference checking of the included papers did not result in studies to be included.

Critical appraisal of these papers is summarized in Table 1.

**Table 1:** Characteristics of the included studies

Author	Line material	Line volume (mL)	Flow rate (mL/h)	Input concentration (U/mL)	Insulin type	Priming methods	Analytical assay	Results Insulin recovery (%)^a^		Conclusions from the authors
								Priming/preconditioning^b^	Non-priming	
Zahid et al. [1]	PVC	0.4	0.1	1	Actrapid 100 U/mL	Non-priming	HPLC-UV	Not investigated	*t*_200 min_ = 54.9 *t*_400 min_ = 65.7 *t*_600 min_ = 74.4 *t*_800 min_ = 79.8 *t*_1000 min_ = 85.1 *t*_1200 min_ = 86.0 *t*_1400 min_ = 90.0	➢ Lower flow rate: more adsorption of insulin to neonatal line ➢ Maximum loss off insulin to neonatal line: at start of infusion, with higher insulin recovery when neonatal line was saturized ➢ After 24 h neither flow rates reached a 100% insulin recovery
			0.5						*t*_200 min_ = 72.8 *t*_400 min_ = 80.2 *t*_600 min_ = 84.4 *t*_800 min_ = 87.7 *t*_1000 min_ = 89.4 *t*_1200 min_ = 91.5 *t*_1400 min_ = 91.5	
Simeon et al. [2]	Not reported	5 inch	0.5	0.5	Humulin R 100	7 mL priming vs non-priming	Lowry protein assay	*t*_0–2 h_ = 55.9 (5.7) *t*_2–4 h_ = 62.3 (6.7) *t*_4–6 h_ = 72.1 (2.1) *t*_6–8 h_ = 71.9 (8.4)	t_0–2 h_ = 67.2 (5.7) *t*_2–4 h_ = 65.8 (4.0) *t*_4–6 h_ = 74.1 (5.4) *t*_6–8 h_ = 78.3 (1.1)	➢ Priming prior to infusion resulted in a more predictable amount of insulin delivered over time ➢ A 2-h delay in insulin recovery with a non-primed line compared to a primed line. ➢ No significant difference in insulin recovery between concentrations of 0.5 and 0.25 U/mL when administered over a primed line.
				0.25				*t*_0–2 h_ = 70.9 (5.8) *t*_2–4 h_ = 63.0 (3.2) *t*_4–6 h_ = 71.4 (5.8) *t*_6–8 h_ = 75.9 (5.6)		
Fuloria et al. [3]	PVC	0.3	0.05	0.2	Novolin R 100 U/mL	Priming vs non-priming	IMx immunoassay	*t*_1 h_ = 71.5 *t*_2 h_ = 77.6 *t*_4 h_ = 69.4 *t*_8 h_ = 88.3 *t*_18 h_ = 85.7 *t*_24 h_ = 93.1	*t*_1 h_ = 16.9 *t*_2 h_ = 10.9 *t*_4 h_ = 27.8 *t*_8 h_ = 55.6 *t*_18 h_ = 54.4 *t*_24 h_ = 95.0	➢ Priming prior to infusion vs non-priming resulted in a higher insulin recovery ➢ Non-priming of lines resulted in problematic low insulin recovery during the first 2 h (up to 8 h) ➢ Higher flow rates resulted in faster insulin recovery in both PE and PVC lines. ➢ A stable higher insulin recovery in PE lines (after 1 h,80%) compared to PVC lines
			0.2					*t*_1 h_ = 43.4 *t*_2 h_ = 85.9 *t*_4 h_ = 92.6 *t*_8 h_ = 92.1 *t*_18 h_ = 98.8 *t*_24 h_ = 100	*t*_1 h_ = 22.6 *t*_2 h_ = 38.6 *t*_4 h_ = 68.1 *t*_8 h_ = 76.7 *t*_18 h_ = 70.9 *t*_24 h_ = 84.2	
	PE	1	0.2					t_1h_ = 80.7 t_2h_ = 77.1 t_4h_ = 72.5 t_8h_ = 59.3 t_18h_ = 76.5 t_24h_ = 49.4	*t*_1 h_ = 20.2 *t*_2 h_ = 13.1 *t*_4 h_ = 22.8 *t*_8 h_ = 65.8 *t*_18 h_ = 82.1 *t*_24 h_ = 85.6	
Hewson et al. [4]	PVC	4	1	0.2	Actrapid 200 mU/mL	Non-priming, 60 min pre-conditioning, 20 mL priming, albumin 2.4 g%, Preconditioning and priming	Radioimmunoassay	*60 min preconditioning* *t*_0.05 h_ = 40.4 *t*_0.25 h_ = 47.1 *t*_0.50 h_ = 39.1 *t*_1 h_ = 49.7 *t*_2 h_ = 64.9 *t*_6 h_ = 79.1 *t*_22 h_ = 73.3	*t*_0.05 h_ = 34.4 *t*_0.25 h_ = 73 *t*_0.50 h_ = 76 *t*_1 h_ = 75.5 *t*_2 h_ = 71.8 *t*_6 h_ = 71.4 *t*_22 h_ = 80	➢ Preconditioning followed by priming of the neonatal line or adding of albumin to insulin solution resulted in the highest insulin recovery
								*20 mL priming* *t*_0.05 h_ = 60.4 *t*_0.25 h_= 52.9 *t*_0.50 h_ = 58.2 *t*_1 h_ = 66.67 *t*_2 h_ = 82.2 *t*_6 h_ = 84 *t*_22 h_ = 83.1		
								*albumin 2.4 g%* *t*_0.05h_ = 34.4 *t*_0.25 h_ = 73 *t*_0.50 h_ = 76 *t*_1 h_ = 75.5 *t*_2 h_ = 71.8 *t*_6h_ = 71.4 *t*_22 h_ = 80		
								*Preconditioning and priming* *t*_0.05 h_ = 51.2 *t*_0.25 h_ = 53.6 *t*_0.50 h_ = 52.4 *t*_1 h_ = 53.6 *t*_2 h_ = 64 *t*_6 h_ = 64.4 *t*_22 h_ = 62.8		
			0.5	0.2				*60 min preconditioning* *t*_0.05 h_ = 58.8 *t*_0.25 h_ = 68.0 *t*_0.50 h_ = 67.6 *t*_1 h_= 64.4 *t*_2 h_ = 60.4 *t*_6 h_ = 82.0 *t*_22 h_ = 54.8	*t*_0.05 h_ = 26.3 *t*_0.25 h_ = 23.3 *t*_0.50 h_ = 15.8 *t*_1 h_ = 15.0 *t*_2 h_ = 15.8 *t*_6 h_ = 48.3 *t*_22 h_ = 64.6	
			1	0.05				*Preconditioning and priming* *t*_0.05 h_ = 24.4 *t*_0.25 h_ = 12.0 *t*_0.50 h_ = 10.4 *t*_1 h_ = 11.6 *t*_2 h_ = 15.6 *t*_6 h_ = 52.4 *t*_22 h_ = 62.8	*t*_0.05 h_ = 33.3 *t*_0.25 h_ = 11.7 *t*_0.50 h_ = 0.8 *t*_1 h_ = 3.3 *t*_2 h_ = 0.4 *t*_6 h_ = 0.8 *t*_22 h_ = 52.1	
			0.5	0.05						
Goldberg et al. [5]	PVC	100 inch	N.A.	1	Novolin R 100	0 mL priming 10 mL priming 20 mL priming 30 mL priming 40 mL priming 50 mL priming	Double antibody insulin radioimmunoassay	*t*_0 h_ = 84.2 *t*_0 h_ = 93.4 *t*_0 h_= 96.6 *t*_0 h_ = 98.7 t_0h_ = 97.9 *t*_0 h_ = 100	Not investigated	➢ 20 mL priming volume is sufficient to minimalize effect of insulin adsorption loss in neonatal line
Thompson et al. [6]	polypropylene	100 inch	N.A.	0.1	Novolin R U-100	Preconditioning (different times 0–1 h)	Double antibody radioimmunoassay	*Preconditioning* *t*_0 h_ = 82 (7) *t*_0.25 h_ = 84 (2) *t*_0.5 h_ = 79 (5) *t*_1 h_ = 83 (6)		➢ Preconditioning time does not affect insulin adsorption Higher concentration of insulin, higher recovery
				1				*t*_0 h_ = 92 (10) *t*_0.25 h_ = 97 (12) *t*_0.5 h_ = 103 (9) *t*_1 h_ = 105 (7)		
				10				*t*_0 h_ = 98 (11) *t*_0.25 h_ = 101 (6) *t*_0.5 h_ = 97 (11) *t*_1 h_ = 103 (8)		

*HPLC-UV* high-performance liquid chromatography-ultraviolet, *N.A.* not applicable, *PE* polyethylene, *PVC* polyvinyl chloride

^a^Values not reported in a table or in the text were extracted from the graphs using plotdigitizer

^b^Priming, unless otherwise specified in the table

Besides the original studies in Table 1, Knopp et al. [7, 8] performed two studies by collecting in vitro adsorption data from literature (all studies included in Table 1) to develop an adsorption model. This model served to calculate the insulin recovery, total insulin adsorption capacities of polyethylene (PE) and polyvinyl chloride (PVC) lines at clinical relevant flow rates, and concentrations. The authors concluded that priming the line with insulin solution prior to infusion could reduce insulin adsorption. By priming a line liquid (e.g., insulin solution) is forced through the line to remove all air within a relative short period of time (few minutes). The advantage of priming is, in this case, that insulin can partly adsorb to the line before being administered to the newborn. When the initial insulin dosing is administered to the newborn, less insulin will adhere to the line and stable insulin dosing will be reached faster. A limitation of this method is that faster flow rates (60–600 mL/h vs 0.1–5 mL/h) seem to result in a general lower adsorption (lack of time for the priming solution to attach to the material). During preconditioning of the line, the line is soaked for a certain amount of time, and overall a longer period of time then for priming namely 20–60 min, with insulin solution prior to infusion. This enables insulin to adhere to the line and could diminish adsorption during insulin administration to the newborn and is more effective combined with priming than priming alone. However, it requires a delay of around 20–60 min in the initiation of insulin therapy within neonates. In addition, it is an effective way to diminish insulin adsorption when albumin is administered to the insulin solution. However, the authors mentioned that administration of albumin could result in health concerns. Therefore, Knopp et al. recommend to precondition the neonatal lines prior to infusion. No method, however, is capable of providing a recovery of 100% and focus is advised during the first 1–6 h after insulin administration before a stable insulin dose is achieved [7, 8].

One study was retrieved in which the researcher tried to establish a relation between their in vitro study and clinical practice [3]. To relate insulin flow rate and blood glucose levels over time, a medication dossier of 13 extreme low birth weight (ELBW) neonates with hyperglycaemia, treated with continuous low dosing of insulin through a neonatal infusion line which was not primed, was assessed. This study showed a 14–24-h delay in blood glucose normalization despite steps wise increase in insulin infusion rates. Thereafter, blood glucose levels quickly decreased, despite a decrease in insulin infusion rate. This suggest that other factors than insulin dosing contributes to the time delay in the clinical glucose reaction. The authors hypothesized that this time delay was related to the adsorption of insulin to the neonatal line.

## Discussion

Insulin is used to treat neonatal hyperglycaemia when blood glucose concentrations are consistently high, and to treat neonatal diabetes. In preterm babies, the prevalence of hyperglycemia is between 50 and 60% of infants [9]. This is caused by their abnormal response to intravenous glucose administration, at times enteral feeding is insufficient for normal metabolism and growth. Risk of hyperglycemia includes dehydration, retinopathy of prematurity and mortality (the latter two without proof of causality). In this context, the evidence of the benefit of insulin therapy for hyperglycemia is lacking. A major risk of insulin therapy is hypoglycemia which is a risk factor for neurological damage. The balance weighing these known and unknown factors for the individual patient is difficult to set. There is some debate about insulin adsorption to infusion material at initiation of insulin therapy. Theoretically, insulin adsorption may cause an unexpected fall of the glucose level at the time the adsorption has reached saturation. A more stable and precise administration of the intended insulin dose from the start of therapy may reduce the risk of hypoglycaemia.

Insulin adsorption to infusion material can influence blood glucose control. Adsorption of insulin to the line can mimic pseudo-insulin resistance of a newborn. Higher insulin dosing combined with saturation of the line with insulin could result in overdosing and hypoglycaemia. The aim of this review was, through searching the existing literature, to investigate if intravenous administration of insulin should be proceeded by priming of the intravenous system, adding of albumin, or non-priming to get a stable insulin dose in preterm babies with hyperglycaemia on the NICU Within this literature search we focused on experimental insulin adsorption data (in vitro data).

**Fig. 1:**
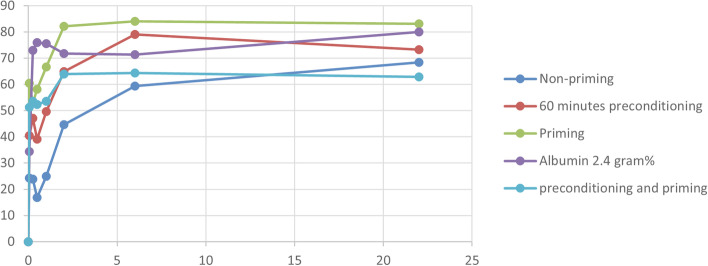
Insulin recovery (%) versus time (hours)

From the in vitro studies (Table 1) can be concluded that various variables can influence the insulin recovery such as administration rate and line material. Most studies investigated the effect of priming prior to infusion versus non-priming [2–4], showing priming prior to infusion resulted in a higher insulin recovery compared to non-priming. It should be noted that only one study [4] compared all scenarios—non-priming of the line, priming prior to infusion, and adding albumin—with each other. In Fig. 1, these three scenarios are shown. It should be noted that the remaining study characteristics (administration rate, insulin concentration) are the same, so the three scenarios could be compared directly.

When the translation is made from in vitro results to the clinical practice, we conclude that preconditioning followed by priming results in a smoother insulin delivery profile. Knopp et al. mentioned a delay of around 20–60 min in the initiation of insulin therapy within neonates with preconditioning and priming. However, in clinical practice acute administration of insulin to a neonate with hyperglycaemia is, in most cases, not urgent. Therefore, a delay of 20–60 min, resulting from preconditioning and priming the line, is acceptable. Besides preconditioning combined with priming, addition of albumin resulted likewise in a high recovery of insulin, even within the first hours after insulin administration. Although more evidence arises that human substances, among which albumin, have adverse effects. Therefore some hesitation exists to administer albumin to the neonatal population [10, 11]. It has to be noted that the exact prevalence of adverse events and the nature of these events attributed to human substances are to date unknown. A study of Curely et al. shows that in 660 infants a related adverse event occurred that can be attributed to the human transfusion product [11]. When comparing priming with non-priming of the line, priming prior to infusion results in a higher percentage of insulin recovery over time. It has to be noted that priming during the first hours did not result in complete recovery of insulin over the line; a loss of 20% has to be taken into account. Monitoring on possible overdosing of insulin during the first 3–6 h after administration due to adsorption of insulin to the line (despite priming) is necessary.

A limitation of the in vitro studies currently performed is that they primarily focussed on the question if insulin administration should be proceeded by priming, adding albumin or non-priming to get a sufficient insulin steady-state concentration and which variables influence the adsorption process of insulin on the line. It is yet unknown which exact concentration or which appropriate priming volume is needed to reach a sufficient insulin steady-state concentration.

## Conclusion

To get a stable insulin dose in preterm newborns with hyperglycaemia on the NICU, intravenous administration of insulin should be proceeded by combining preconditioning with priming of the intravenous system

## Data Availability

No data are available to share.
